# Contribution of Tissue Inflammation and Blood-Brain Barrier Disruption to Brain Softening in a Mouse Model of Multiple Sclerosis

**DOI:** 10.3389/fnins.2021.701308

**Published:** 2021-08-23

**Authors:** Rafaela Vieira Silva, Anna S. Morr, Susanne Mueller, Stefan Paul Koch, Philipp Boehm-Sturm, Yasmina Rodriguez-Sillke, Désirée Kunkel, Heiko Tzschätzsch, Anja A. Kühl, Jörg Schnorr, Matthias Taupitz, Ingolf Sack, Carmen Infante-Duarte

**Affiliations:** ^1^Charité - Universitätsmedizin Berlin, Corporate Member of Freie Universität Berlin and Humboldt-Universität zu Berlin, Institute of Medical Immunology, Berlin, Germany; ^2^Charité - Universitätsmedizin Berlin, Einstein Center for Neurosciences Berlin, Berlin, Germany; ^3^Charité - Universitätsmedizin Berlin, Corporate Member of Freie Universität Berlin and Humboldt-Universität zu Berlin, Department of Radiology, Berlin, Germany; ^4^Charité - Universitätsmedizin Berlin, Corporate Member of Freie Universität Berlin and Humboldt-Universität zu Berlin, Department of Experimental Neurology and Center for Stroke Research, Berlin, Germany; ^5^Charité - Universitätsmedizin Berlin, NeuroCure Cluster of Excellence and Charité Core Facility 7T Experimental MRIs, Berlin, Germany; ^6^Berlin Institute of Health at Charité - Universitätsmedizin Berlin, Flow & Mass Cytometry Core Facility, Berlin, Germany; ^7^Charité - Universitätsmedizin Berlin, Corporate Member of Freie Universität Berlin and Humboldt-Universität zu Berlin, Berlin, Germany; ^8^Charité - Universitätsmedizin Berlin, Corporate Member of Freie Universität Berlin and Humboldt-Universität zu Berlin, ECRC Experimental and Clinical Research Center, Berlin, Germany

**Keywords:** magnetic resonance elastography, Eu-VSOP, gadolinium, neuroinflammation, experimental autoimmune encephalomyelitis, BBB disruption, multiple sclerosis

## Abstract

Neuroinflammatory processes occurring during multiple sclerosis cause disseminated softening of brain tissue, as quantified by *in vivo* magnetic resonance elastography (MRE). However, inflammation-mediated tissue alterations underlying the mechanical integrity of the brain remain unclear. We previously showed that blood-brain barrier (BBB) disruption visualized by MRI using gadolinium-based contrast agent (GBCA) does not correlate with tissue softening in active experimental autoimmune encephalomyelitis (EAE). However, it is unknown how confined BBB changes and other inflammatory processes may determine local elasticity changes. Therefore, we aim to elucidate which inflammatory hallmarks are determinant for local viscoelastic changes observed in EAE brains. Hence, novel multifrequency MRE was applied in combination with GBCA-based MRI or very small superparamagnetic iron oxide particles (VSOPs) in female SJL mice with induced adoptive transfer EAE (*n* = 21). VSOPs were doped with europium (Eu-VSOPs) to facilitate the *post-mortem* analysis. Accumulation of Eu-VSOPs, which was previously demonstrated to be sensitive to immune cell infiltration and ECM remodeling, was also found to be independent of GBCA enhancement. Following registration to a reference brain atlas, viscoelastic properties of the whole brain and areas visualized by either Gd or VSOP were quantified. MRE revealed marked disseminated softening across the whole brain in mice with established EAE (baseline: 3.1 ± 0.1 m/s vs. EAE: 2.9 ± 0.2 m/s, *p* < 0.0001). A similar degree of softening was observed in sites of GBCA enhancement i.e., mainly within cerebral cortex and brain stem (baseline: 3.3 ± 0.4 m/s vs. EAE: 3.0 ± 0.5 m/s, *p* = 0.018). However, locations in which only Eu-VSOP accumulated, mainly in fiber tracts (baseline: 3.0 ± 0.4 m/s vs. EAE: 2.6 ± 0.5 m/s, *p* = 0.023), softening was more pronounced when compared to non-hypointense areas (percent change of stiffness for Eu-VSOP accumulation: −16.81 ± 16.49% vs. for non-hypointense regions: −5.85 ± 3.81%, *p* = 0.048). Our findings suggest that multifrequency MRE is sensitive to differentiate between local inflammatory processes with a strong immune cell infiltrate that lead to VSOP accumulation, from disseminated inflammation and BBB leakage visualized by GBCA. These pathological events visualized by Eu-VSOP MRI and MRE may include gliosis, macrophage infiltration, alterations of endothelial matrix components, and/or extracellular matrix remodeling. MRE may therefore represent a promising imaging tool for non-invasive clinical assessment of different pathological aspects of neuroinflammation.

## Introduction

Multiple sclerosis (MS) is a chronic autoimmune disease in which myelin-autoreactive immune cells gain access to the central nervous system (CNS) *via* the blood-brain barrier (BBB) and the blood-cerebrospinal fluid barrier (Alvarez et al., 2011). This infiltration results in the formation of multiple focal lesions that involve both white and gray matter and contribute to demyelination and neurodegeneration. Demyelinating plaques are seen as hyperintense areas by T2-weighted magnetic resonance imaging (MRI), whereas inflammation-associated BBB leakage is revealed by hyperintensity in contrast agent-based T1-weighted images (Thompson et al., 2018).

Due to their intrinsic magnetic properties, gadolinium-based contrast agents (GBCAs) have long been used as contrast agents for MRI. GBCAs induce signal changes in T2, T2^∗^ and T1-weighted MRI by affecting the relaxation time of water protons in biological tissues. The appearance of hyperintense widespread Gd-enhanced areas has become the standard MRI marker of loss of BBB integrity on T1-weighted MRI. Because BBB leakage was found to predict the formation of new non-focal white matter lesions, GBCA enhancement reflecting inflammatory activity is widely used in MS diagnosis (Lassmann, 2008; Zéphir, 2018). However, studies on the potential accumulation of Gd into the tissue, including the CNS, after repeated administration of GBCAs, in particular linear GBCAs, raised concerns about their safety (Layne et al., 2018). Furthermore, a discrepancy between the presence of Gd-enhancing lesions and clinical manifestations in both, MS and its animal model, the experimental autoimmune encephalomyelitis (EAE), has been reported (Lassmann, 2008) (Wuerfel et al., 2007, 2010; Tysiak et al., 2009; Wang et al., 2019). This discrepancy, referred to as a clinical-radiological paradox, motivates the quest for reliable imaging tools in neuroinflammation.

An evolving imaging strategy for visualizing neuroinflammation is based on the application of very small iron oxide particles (VSOPs) (Tysiak et al., 2009; Millward et al., 2013). VSOPs have a hydrodynamic diameter of only 7 nm and are stabilized with a citrate coating (Taupitz et al., 2000; Wagner et al., 2002). The iron causes strong susceptibility changes, which give rise to hypointense areas in T1-, T2- and T2^∗^-weighted MR images, but appears to be best visualized by T2^∗^weighted MRI (Tysiak et al., 2009; Millward et al., 2013, 2019). Previous work in EAE has demonstrated that VSOPs administered *in vivo* intravenously can be phagocytosed locally or transported within cells to areas of inflammation and/or appear freely diffused in the brain parenchyma or bound to endothelial cells (Tysiak et al., 2009; Millward et al., 2013, 2019; Berndt et al., 2017). Therefore, VSOPs do not only visualize BBB breakdown in areas of Gd-enhancement but additionally detect other inflammatory mechanisms that were not detected by conventional GBCA-enhanced MRI, such as myeloid cell infiltrates, changes in the extracellular matrix (ECM) or alteration of the endothelial glycocalyx (Tysiak et al., 2009; Millward et al., 2013, 2019; Berndt et al., 2017).

At the same time, magnetic resonance elastography (MRE) (Muthupillai et al., 1995) has evolved as a non-invasive tool for detecting inflammation-associated pathologies (Muthupillai et al., 1995; Plewes et al., 1995; Schregel et al., 2012; Hiscox et al., 2016). It is already well documented that neuroinflammation modifies the viscoelastic properties of neuronal tissue, reducing stiffness throughout larger brain areas (Wuerfel et al., 2010; Streitberger et al., 2012) (Riek et al., 2012; Millward et al., 2015; Fehlner et al., 2016; Wang et al., 2020). MRE uses harmonic vibrations to transmit shear waves into the tissue of interest (Manduca et al., 2021). Wave propagation is detected using motion-sensitive MRI, from which viscoelastic parameters such as stiffness (magnitude shear modulus or shear wave speed) and fluidity (viscosity, dispersion angle or loss angle of the shear modulus) are derived (Hirsch et al., 2017). We previously demonstrated in the active EAE model that MRE does not appear to correlate with GBCA enhancement in MRI. However, in this study, we could not provide an accurate regional analysis due to the limitation of the MRE tools (Wang et al., 2020). The recent development of multifrequency MRE with tomoelastography postprocessing in small animal models fosters high-resolution MRE of the mouse brain with multiple imaging slices that reveal deeper anatomical details (Bertalan et al., 2019), enabling a more accurate investigation of the mechanisms underlying brain softening in inflamed areas.

Therefore, in this study, we aimed at uncovering how neuroinflammatory processes such as blood-brain barrier leakage and focal inflammation are associated with changes in the mechanical properties detected by multifrequency MRE. To optimize the study, here we use a model of adoptive transfer EAE, which contrary to the active EAE used in Wang et al., 2020 (Wang et al., 2020) develops numerous and large brain lesions. We hypothesize that novel tomoelastography sensitively detects mechanical changes in sites of intense neuroinflammation that are revealed by VSOP-deposits. Those sites are known to be characterized by enhanced myeloid cell activity (Millward et al., 2013), alterations of the glycocalyx on barrier cells (Berndt et al., 2017) and ECM remodeling (Wang et al., 2020). To test our hypothesis, we combined *in vivo* viscoelasticity measured by multifrequency MRE with both, GBCA- and VSOP- MRI, in the adoptive transfer EAE model. We used VSOPs doped with europium (Eu) in order to improve histological detection of the particles in biological tissues, without the interference of endogenous iron (de Schellenberger et al., 2017).

## Materials and Methods

### Animals and Adoptive Transfer EAE Induction

All animal experiments were conducted in accordance with national and institutional guidelines for the care and use of laboratory animals and with directive 2010/63/EU of the European Parliament and of the Council of 22 September 2010 and were approved by the Berlin State Office for Health and Social Affairs (LAGeSo, registration number G106/19).

Adoptive transfer EAE was induced by transfer of myelin proteolipid protein (PLP)-reactive lymphocytes into two cohorts of adult female SJL mice, 9–12 weeks old (*n* = 21 mice in total; Janvier, SAS, France). EAE is more effectively induced in females than in male animals (Miller and Karpus, 2007), therefore in all our previous studies and also in this study we included only female mice in the experiments. To obtain PLP-reactive lymphocytes, a donor group of mice (*n* = 22) was actively immunized with 200 μg of the myelin peptide PLP139-151 emulsified with 200 μl complete Freund’s adjuvant (Thermo Fischer Scientific, United States) and 800 μg Mycobacterium tuberculosis H37Ra (Difico, United States) as previously described (Millward et al., 2013). Additionally, 250 ng pertussis toxin (List Biological Laboratories, United States) was injected intraperitoneally on days 0 and 2. On day 10 post-immunization, animals were sacrificed, inguinal and axillary lymph nodes were collected, and the cells were cultured as previously described (Plewes et al., 1995). After 4 days in culture with RPMI 1640 medium (supplemented with 2 mM L-glutamine, 100 units/ml penicillin, 100 μg/ml streptomycin and 10% fetal bovine serum) (Gibco, Thermo Fischer Scientific) containing 12.5 μg/ml PLP, lymph node cells were harvested, and 30 million cells were injected intraperitoneally into each recipient mouse. After immunization, mice were monitored daily for signs of disease, which were scored as follows: 0—no sign, 0.5—tail paresis, 1—tail paresis and/or plegia and righting reflex weakness; 2—hind limb paresis; 3—paraplegia; 4—paraplegia with forelimb weakness or paralysis; 5—moribund or dead animal. To comply with animal welfare guidelines, all mice with a score greater than 3 or with atypical signs of EAE were euthanized and removed from the study before the second T2^∗^-weighted MRI (*n* = 8).

### *In vivo* Scans and Experimental Set-Up

**FIGURE 1:**
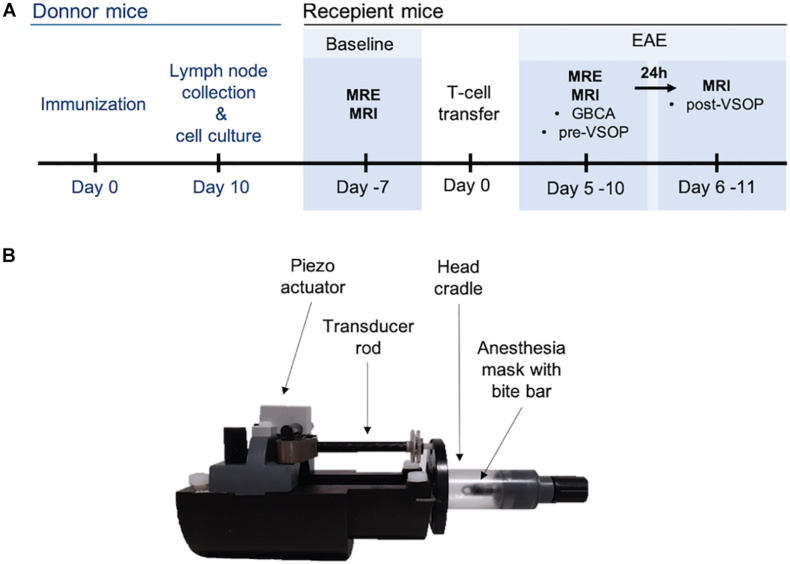
Experimental set-up. **(A)** Timeline depicting induction of adoptive transfer EAE and *in vivo* scans at baseline, before immunization, and after establishment of EAE. **(B)** Custom-made animal holder. A piezo actuator generates the mechanical waves, which are transmitted *via* the transducer to the head cradle and into the skull of the mouse.

*In vivo* scans were performed at two timepoints, prior to EAE induction (baseline control) and after EAE signs were established, i.e., animals showed at least partial hind limb paresis (score 1.75) (Figure 1A). For each single animal, EAE scan was compared to the corresponding baseline scan, while imaging post-contrast was compared to the corresponding pre-contrast image. In EAE animals, MRE was first performed followed by pre-contrast MRI with a T1-weighted imaging, a T2-weighted sequence to acquire an anatomical image, and a pre-contrast T2^∗^-weighted sequence. Thereafter, GBCA (0.2 mmol/kg, Magnevist, Bayer-Schering AG) was administered *via* the tail vein, and post-contrast T1-weighted images were acquired, At the end of the scans, 0.2 mmol/kg Eu-VSOPs [batch RH030812 Eu-R; c(Fe) = 0.134 mol/L] were intravenously injected into the tail vein, and 24 h later, a T2^∗^-weighted sequence was acquired to visualize particle accumulation. The applied Eu-VSOP and Magnevist doses as well as the time between injections and scans were defined based on our previous studies (Wuerfel et al., 2007; Tysiak et al., 2009; Millward et al., 2013, 2019; Wang et al., 2020). No GBCA or Eu-VSOPs were administered at baseline. Eu-VSOPs were produced and provided by the Experimental Radiology working group of the Department of Radiology, Charité - Universitätsmedizin Berlin as described previously (de Schellenberger et al., 2017).

MRI and MRE examinations were performed in a preclinical 7 Tesla MRI scanner (BioSpec, Bruker, Ettlingen, Germany) running with ParaVision 6.1 software. All scans were acquired with a 20-mm diameter 1H-RF quadrature volume coil (RAPID Biomedical, Würzburg, Germany). For acquisitions, mice were placed on a custom-built animal holder (Figure 1B) and anesthetized with 1.5–2.0% isoflurane in 30% O_2_ and 70% N_2_O administered *via* an anesthesia mask during continuous respiratory monitoring using a pressure-sensitive pad placed on the thorax (Small Animal Instruments Inc., Stony Brook, NY, United States). Body temperature was kept constant by circulating water through warming pads integrated into the animal holder, and body temperature was monitored using a rectal probe.

#### MRI

Coronal anatomical images were acquired using a T2-weighted 2D-RARE sequence with repetition time (TR) = 3,500 ms, effective echo time (TE) = 33 ms, echo spacing (DTE) = 11 ms, RARE factor = 8, 4 averages, 32 contiguous slices with a slice thickness of 0.5 mm, field of view (FOV) = 18 mm × 18 mm, matrix size MTX = 180 × 180, in-plane resolution 0.1 mm × 0.1 mm × 0.5 mm, bandwidth BW = 34,722 Hz, and total acquisition time TA = 5:08 min). GBCA-enhanced images were acquired using a T1-weighted RARE sequence with TR = 800 ms, TE = 6.5 ms, (DTE) = 6.5 ms, RARE factor = 2, 6 averages, BW = 75,000 Hz, and the same geometry as for the T2w scan resulting in a total acquisition time of TA = 7:12 min. To visualize Eu-VSOP accumulation, a T2^∗^-weighted FLASH sequence was used with TR = 400 ms, TE = 2.5 ms, flip angle = 30 deg, 3 averages, BW = 29,762 Hz, and the same geometry as for the T2w scan with a total acquisition time of TA = 2:24 min.

#### MRE

**FIGURE 2:**
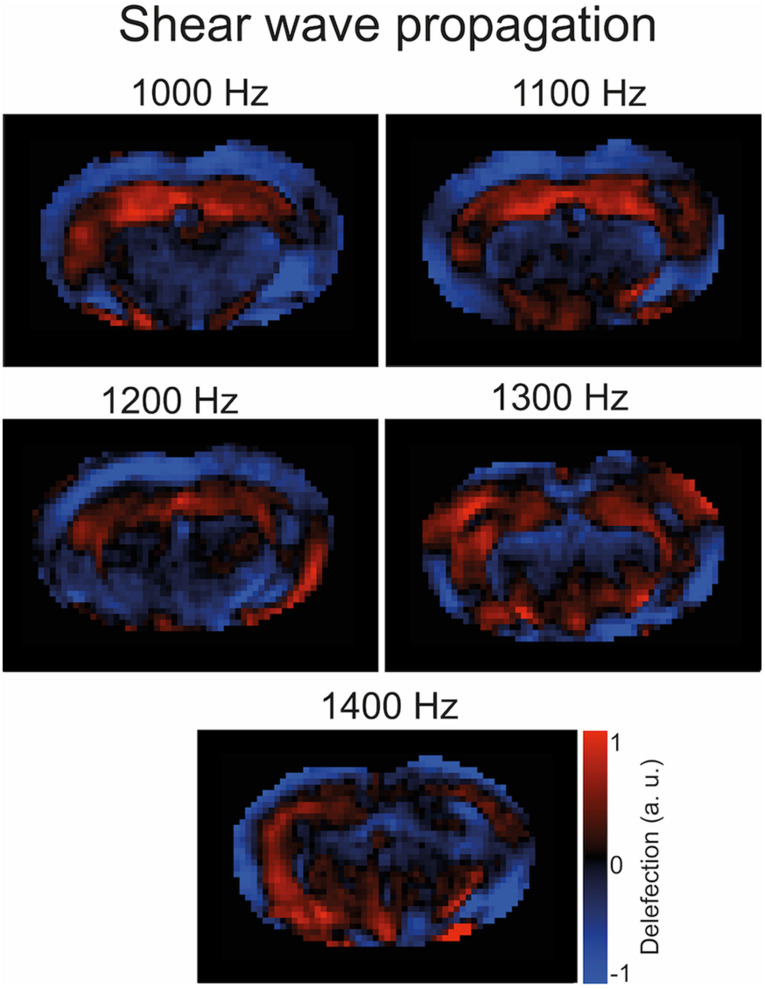
Representative wave images. Propagation of shear waves through the mouse brain after *k*-MDEV inversion (1 slice, 1 component, 5 frequencies, and no directional filters applied). The image was taken from a mouse after immunization.

To induce shear waves in the mouse brain, vibrations were generated with a custom-made driver system using a non-magnetic piezoceramic actuator. Vibrations were transmitted *via* a transducer rod to the head cradle and into the skull of the anesthetized and fixated mouse (Figure 1B). The MRE technique used in this study has been described in more detail elsewhere (Bertalan et al., 2019). In short, multifrequency MRE was performed, and wave images (Figure 2) were acquired using 5 frequencies (1,000, 1,100, 1,200, 1,300, and 1,400 Hz). In the bregma areas −2.84 mm to 0.23 mm, 7 coronal slices with a slice thickness of 0.8 mm and a 0.18 mm × 0.18 mm in-plane resolution were acquired. We limited our analysis to this area for consistency with previous studies using tomoelastography (Bertalan et al., 2019, 2020; Guo et al., 2019), as well as to reduce scan time while focusing on multifrequency studies. Further imaging parameters were: TA = 9 min, TE = 53 ms, TR = 4,000 ms, FOV = 16.2 mm × 10.8 mm, and matrix size = 90 × 60.

For analysis, tomoelastography postprocessing (Tzschätzsch et al., 2016) based on multifrequency wave-number analysis was used to derive shear wave speed (c in m/s) as a surrogate marker of stiffness. In addition, Laplacian-based direct multifrequency inversion (Hirsch et al., 2017) was used to recover the phase angle of the complex shear modulus (ϕ in rad, also known as loss angle) as a measure of the solid-fluid behavior of biological tissues. Averaged c- and ϕ-maps showing the brain area covered by MRE (from bregma −2.84 to 0.23 mm) are provided in Supplementary Figure 1.

### Data Analysis

#### Registration to the Allen Brain Atlas

ANTx, a customized MATLAB toolbox (latest version available under^1^), was used for MRI and MRE image registration, as described elsewhere (Koch et al., 2019). In short, for MRI, T2-weighted RARE images, pre- and postcontrast T1-weighted RARE, and T2^∗^-weighted FLASH images were transferred into the Allen mouse brain atlas space (Allen Institute for Brain Science, United States) using ELASTIX^2^ (Klein et al., 2009). Next, the pre- and postcontrast T1-weighted RARE and the pre- and postcontrast T2^∗^-weighted FLASH images were co-registered and resliced to the T2-weighted RARE image using affine transformation.

For image registration of the MRE parameter maps to the brain atlas, the individual magnitude images and the parameter maps, c and ϕ, were first 3D-coregistered and 2D-slice wise registered to the to the T2-weighted TurboRare images using affine non-linear b-spline transformation. Finally, the image transformation found for MRI was used to transform MRE images into the Allen mouse brain atlas space (Guo et al., 2019). All acquired 7 MRE slices were interpolated to 215 slices of the reference atlas, generating approx. 58 corresponding MRE slices. Consequently, all acquired scans were aligned to the Allen mouse brain atlas, making it possible to locate and compare identified areas of Gd-enhancement and Eu-VSOP accumulation between the different imaging modalities.

#### Generation of Masks

Masks corresponding to areas of Gd-enhancement and Eu-VSOP accumulation were manually drawn on registered postcontrast T1-weighted and T2^∗^-weighted images by two experienced researchers using the ANALYZE 10.0 program (Biomedical Imaging Resources Mayo Clinic). For masks depicting Gd-enhancement, maps showing percentage changes in signal intensity (SI) were calculated by subtracting the postcontrast from the precontrast T1-weighted image using Image J 1.52e (^3^ National Institutes of Health, United States). Eu-VSOP masks were drawn on the postcontrast T2^∗^-weighted images. Then, all masks were registered to the Allen mouse brain atlas using ELASTIX.

As the sites of particle accumulation appear as focal hypointense areas, and the current MRE resolution is 0.18 mm × 0.18 mm, it was necessary to dilate the Eu-VSOP masks by two pixels using Matlab to reduce effects of single-pixel artifacts [Version 9.7 (R2019b); Natick, Massachusetts: The MathWorks Inc.]. Eu-VSOP areas were excluded from GBCA masks and vice versa in order to obtain masks corresponding solely to areas with either Gd-enhancement or Eu-VSOP accumulation. Individual ventricle masks, manually drawn on registered MRE magnitude images, were excluded from the GBCA and Eu-VSOP masks to eliminate the ventricles from the maps, as mechanical wave propagation is not ensured in liquid-filled spaces. In order to establish MRE parameter maps of areas with alterations detected exclusively by one of the MRI modalities (either by GBCA or Eu-VSOP), areas marked in both, GBCA-based and Eu-VSOP-based MRI, were disregarded. Mean values for stiffness and fluidity were obtained by overlaying the masks on the MRE parameter maps (c-map and ϕ-map).

Gadolinium-based contrast agent masks generated from percentage change T1-weighted images and Eu-VSOP masks drawn on (unregistered) T2^∗^-weighted images were used to create incidence maps using the ANTx toolbox in order to illustrate the prevalence of inflammation detected by either GBCA or Eu-VSOPs.

#### Statistical Analysis

Paired *t*-tests were applied to shear wave speed (c) and phase angle (ϕ) mean values of mask obtained from the MRE parameter maps using GraphPad Prism 9.0 (GraphPad software, La Jolla, CA, United States). Normality was tested using the D’Agostino-Pearson test. If one of the two compared groups did not pass the normality test, the Wilcoxon matched-pairs signed rank test was used. Differences were considered to be statistically significant for *p*-values < 0.05.

### Histology and Imaging Mass Cytometry

Imaging Mass Cytometry^TM^ (IMC) was used to identify the surroundings of the particles within the inflamed tissue by using lanthanide-tagged antibodies and the Eu incorporated into the nanoparticle cores to label VSOPs. Therefore, after the last MRI scan, animals were sacrificed with an overdose of ketamine/xylazine and immediately transcardially perfused with 4% paraformaldehyde (PFA) (Carl Roth^®^, Germany). In addition, a healthy mouse without Eu-VSOP administration was used as control. The brains were removed and immersed in 4% PFA for 24 h at 4°C prior to paraffin embedding (ROTI^®^ Plast, Germany). Sequential 4-μm sections of samples, corresponding to areas with Eu-VSOP accumulation identified by MRI, were cut, heated at 60°C for at least 1 h, and deparaffinized in m-xylene overnight (Sigma-Aldrich, Germany) for IMC. Following deparaffinization, the sections were re-hydrated in descending ethanol series (Carl Roth^®^, Germany). Then, samples were processed according to the IMC staining protocol for FFPE sections [PN 400322 A3, Fluidigm San Francisco, CA, United States(RSY1)]. The antigen retrieval was performed with a citrate buffer (10 mM sodium citrate, 0.05% Tween 20, pH 6) for 20 min at 96°C. After incubation with isotope-tagged antibodies overnight at 4°C, the sections were stained with the CELL-ID Intercalator-Ir (Fluidigm, United States) for 30 min at room temperature, then washed, air-dried and kept at RT until imaging with the Hyperion Imaging System (Fluidigm, United States).

All antibodies were tagged with a metal isotope using the Maxpar labeling kit according to the manufacturer’s instructions (Fluidigm, United States). A complete list of the panel with a description of the antibodies, isotope tags, and dilutions is available in Supplementary Table 1. Imaging mass cytometry was performed on a CyTOF2/upgraded to Helios specifications coupled to a Hyperion Tissue Imager (Fluidigm), using CyTOF software version 7.0. Prior to ablation, the instrument was tuned according to the manufacturer’s instructions, using the 3-Element Full Coverage Tuning Slide (Fluidigm). The dried slide was loaded into the imaging module and regions of interest were selected for each sample on a preview (panorama). Optimal laser power was determined for each sample to ensure complete ablation of the tissue. Laser ablation was performed at a resolution of 1 μm and a frequency of 200 Hz. Data was stored as MCD files as well as txt files. Original files were opened with MCD viewer (v1.0.560.6), and images were extracted as TIFF files. For visualization, threshold correction was applied to reduce noise followed by post-acquisition processing with ImageJ software (ImageJ 1.48v, United States) using the despeckle and sharpen tools and Gaussian blur filter (kernel width, 0.50 pixels).

## Results

### GBCA and Eu-VSOP Incidence Maps in Adoptive Transfer EAE

Following transfer of myelin-reactive lymphocytes, all animals showed signs of disease reflecting ascending paralysis. On the first day of imaging after EAE establishment, the mean score was 1.97 (±0.76 SD) versus 2.83 (±0.39 SD) on the second day, 24 h after Eu-VSOP injection. According to the animal protocols, 8 animals with atypical signs of EAE or with a score greater than 3 were removed from the study before the second T2^∗^-weighted acquisition.

**FIGURE 3:**
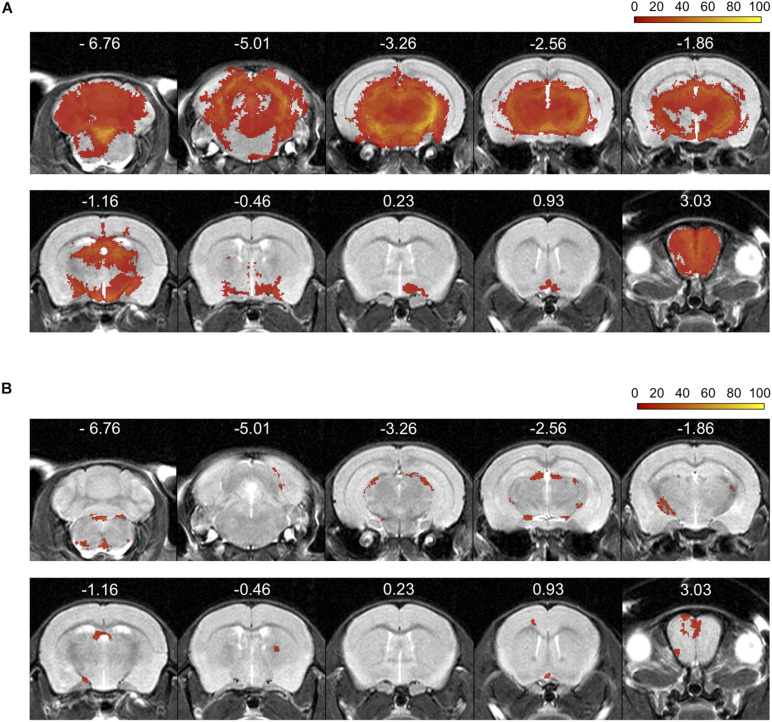
Percentage incidence maps for Gd-enhancement and Eu-VSOP accumulation during EAE. **(A)** Incidence (% of mice affected) of gadolinium distribution across the brain, displaying large diffuse lesions along the cerebral cortex, cerebral nuclei, brain stem, midbrain and fiber tracts (*n* = 19). **(B)** Conversely, Eu-VSOP distribution appears to be characterized by smaller and focal areas of accumulation in the cerebral cortex, cerebral nuclei, brain stem, midbrain and fiber tracts (*n* = 11).

Incidence maps, as percentage of mice affected, were created to visualize the extent and frequency of inflammatory lesions visualized by each of the two contrast agents. The color scale represents the percentage of animals with a mask in these areas (Figure 3). After establishment of the disease, all mice showed Gd- (*n* = 19) and Eu-VSOPs-enhancement (*n* = 11) (bregma coordinates −2.8 to 0.23 mm). Both GBCA and Eu-VSOPs were found throughout the brain. The GBCA-intensity maps, showing large diffuse Gd-enhanced lesions across the brain, indicate that BBB leakage was localized in the same areas among mice, namely near the ventricles. The most affected regions were close to large arteries, such as the left hippocampal artery (bregma coordinate −3.26 mm), present in 80% of the mice (Figure 3A). While the cerebellum and brainstem were particularly affected, enhancement was also found in the hippocampus, thalamus, hypothalamus and midbrain. Specifically within the brain area covered by the MRE scan (bregma coordinates −2.84 to 0.23 mm), GBCA-enhancement was detected within the cerebral cortex, cerebral nuclei, brain stem, midbrain and fiber tracts), as shown in details in the Supplementary Figure 2. Conversely, Eu-VSOP accumulation was more diffuse with a less predictable pattern, with the highest incidence in the vicinity of the basilar artery (bregma coordinate −3.26 mm) and the anterior cerebral artery (bregma coordinate −6.76 mm) of about 40% (Figure 3B). An overview of the Eu-VSOP distribution across the brain can be found in Supplementary Figure 3. Within the brain section covered by MRE, the signal distribution was found spread inside the hippocampus, cerebral nuclei, brain stem, midbrain and fiber tracts. Table 1 additionally shows the incidence of Gd and Eu-VSOP across the whole brain.

**TABLE 1:** Incidence of Gd-enhancement and Eu-VSOP accumulation.

Incidence (%)		

Anatomical label	Gd mask	Eu-VSOP mask
Cerebral cortex	100	72.7
Cerebral nuclei	100	54.5
Brain stem	78.9	90.9
Midbrain	100	63.6
Hindbrain	100	63.6
Cerebellum	100	54.5
Fiber tracts	100	90.9

*Incidence in percent of Gd-enhancement and Eu-VSOP accumulation, per brain region, across the whole brain.*

**FIGURE 4:**
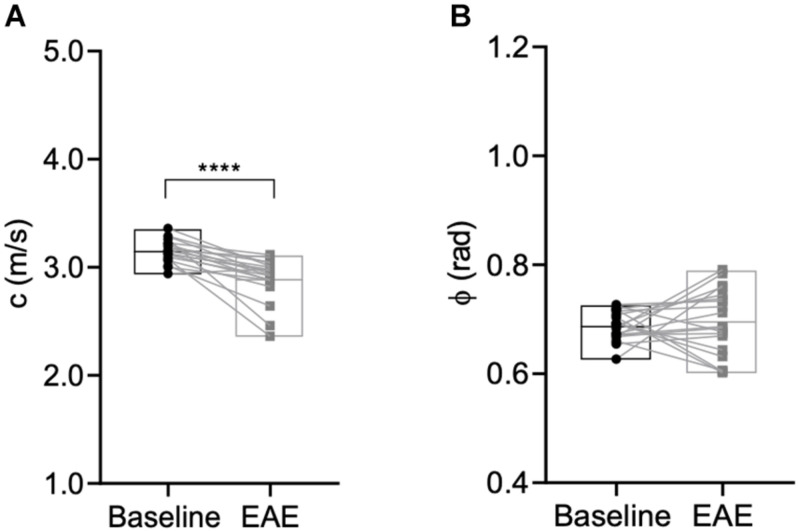
Global brain softening due to inflammation. **(A)** Whole brain stiffness is reduced during inflammation (*p* < 0.0001, Wilcoxon matched-pairs signed rank test), whereas **(B)** fluidity is unaffected (*p* = 0.6070, paired *t*-test). *n* = 19; mean, min/max. ****p ≤ 0.0001.

Next, we assessed the global effect of neuroinflammation on viscoelasticity. Therefore, viscoelastic properties of the whole brain, disregarding the ventricles, were measured at baseline and after establishment of EAE symptoms. A significant softening (reduction in stiffness, i.e., decrease in c in m/s) was observed (*p* < 0.0001, baseline 3.15 ± 0.11 m/s vs. EAE 2.89 ± 0.20 m/s, *n* = 19), whereas the fluidity depicted by the loss angle (ϕ in rad) (*p* = 0.6070, baseline 0.69 ± 0.03 m/s vs. EAE 0.70 ± 0.06 m/s, *n* = 19) was unaffected (Figure 4).

### MRE Detects Tissue Softening in Areas of Gd-Enhancement

**FIGURE 5:**
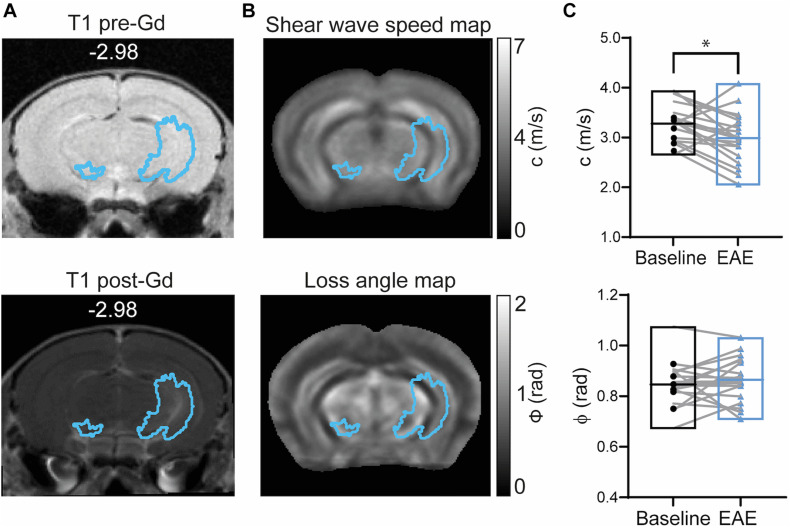
Brain mechanical properties in Gd-enhanced lesions during EAE. **(A)** Representative T1-weighted image acquired before (left) and after GBCA (right) injection (corresponding GBCA mask overlaid) shows Gd-enhancing lesions near the left hippocampal artery. **(B)** GBCA mask overlaid on averaged MRE maps of stiffness (shear wave speed) and fluidity (loss angle). **(C)** Stiffness represented by shear wave speed (c) was reduced during EAE (*p* = 0.0183; paired *t*-test) whereas tissue fluidity (ϕ) was unchanged (*p* = 0.3683; paired *t* test). *n* = 19; mean, min/max; *<0.05. Of note, ventricles are excluded from our analysis since MRE cannot be performed in fluid compartments.

To assess the sensitivity of MRE to measure mechanical alterations in areas of BBB leakage detected exclusively by Gd-enhancement, GBCA masks generated from percentage change images calculated from T1-weighted MR images were overlaid on MRE parameter maps (ϕ and c) as illustrated in Figures 5A,B.

A significant reduction in shear wave speed (c) after EAE establishment was observed compared to baseline (*p* = 0.0183; baseline 3.28 ± 0.38 m/s vs. EAE 2.99 ± 0.51 m/s, *n* = 19), indicating softening of brain tissue, whereas fluidity (phase angle, ϕ) remained unaltered (*p* = 0.3683; baseline 0.85 ± 0.08 rad vs. EAE 0.86 ± 0.09 rad, *n* = 19) (Figure 5C).

### MRE Detects Marked Tissue Softening in Sites of Eu-VSOP Accumulation

**FIGURE 6:**
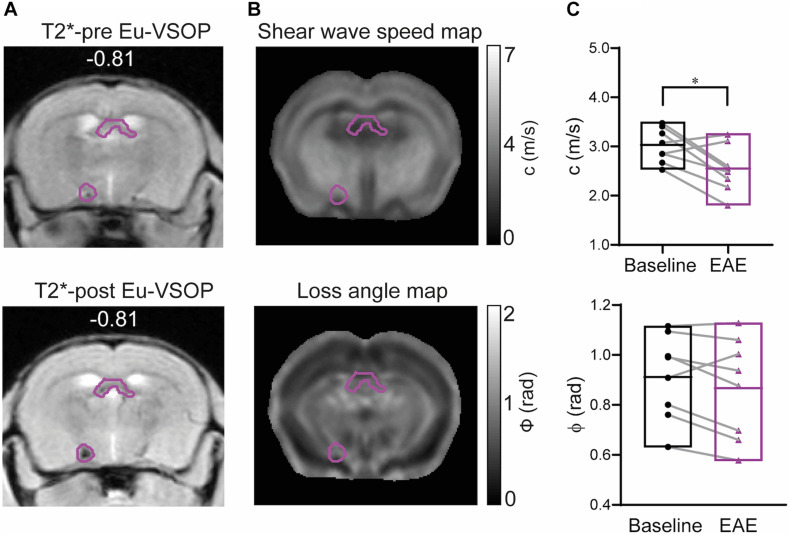
Brain mechanical properties of areas accumulating Eu-VSOPs during EAE. **(A)** Exemplary image of a mask comprising Eu-VSOP accumulation overlaid on T2*-weighted image prior (left) and post Eu-VSOP (right) injection. **(B)** Shear wave speed map (c in m/s) and loss angle map (ϕ in rad). **(C)** There is a significant reduction in stiffness in sites with Eu-VSOP accumulation compared to non-hypointense areas (*p* = 0.0235, paired *t*-test). Fluidity of brain tissue in these areas, marked by ϕ, is not affected (*p* = 0.1168, paired *t*-test). *n* = 8, mean, min/max; *<0.05. Of note, ventricles are excluded from our analysis since MRE cannot be performed in fluid compartments.

Eight of the 11 mice with Eu-VSOP accumulation showed accumulation in regions covered by the MRE scans (bregma −2.84 to 0.23 mm). The masks solely comprising Eu-VSOP accumulation were overlaid on the MRE parameter maps, c and ϕ, as shown in Figures 6A,B. Tomoelastography at baseline and after EAE induction revealed a significant reduction in stiffness (c in m/s) in sites with Eu-VSOP accumulation (within hippocampus, cerebral nuclei, brain stem, midbrain and fiber tracts) (*p* = 0.0235, baseline 3.03 ± 0.39 m/s vs. EAE 2.55 ± 0.47 m/s, *n* = 8); whereas fluidity, depicted by ϕ in rad, was unaffected (*p* = 0.1168, baseline 0.91 ± 0.17 rad vs. EAE 0.87 ± 0.20 rad, *n* = 8) (Figure 6C).

### Sites of Eu-VSOP Accumulation Display Pronounced Softening Compared to the Overall Brain Alterations

**FIGURE 7:**
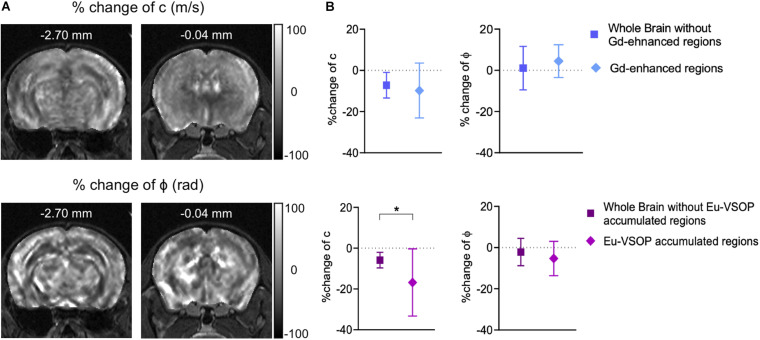
MRE is sensitive to stiffness changes in sites of Eu-VSOP accumulation. **(A)** Representative percentage change maps of c- and ϕ-map. **(B)** Percentage change in areas with Gd enhancement (within cerebral cortex, cerebral nuclei, brain stem, midbrain and fiber tracts) compared to non-enhancing areas revealed no significant difference (top), for c in m/s *p* = 0.3321 (Wilcoxon matched-pairs rank sum test), for ϕ in rad *p* = 0.1246 (paired *t*-test), *n* = 19; percentage change of region with Eu-VSOP accumulation (within cerebral cortex, cerebral nuclei, brain stem, midbrain and fiber tracts) (bottom) compared to non-hypointense areas showed a significant difference for c (*p* = 0.0483, paired *t*-test, mean ± SD, *n* = 8, *<0.05) whereas ϕ was not affected (*p* = 0.2889, paired *t*-test, mean ± SD *n* = 8, *<0.05). Percentage change maps are based on the percentage difference from peak to baseline.

We further investigated whether MRE is sensitive to mechanical changes at sites of inflammation detected by MRI with either GBCA or Eu-VSOPs. For that, the percentage change in stiffness and fluidity in areas with Gd-enhancement or Eu- VSOP accumulation was compared to the rest of the brain (Figure 7A). Our analysis revealed no significant difference in the percentage change between sites with Gd-enhancement (within cerebral cortex, cerebral nuclei, brain stem, midbrain and fiber tracts) compared to non-enhancing areas for either stiffness (*p* = 0.3321, Gd-enhanced areas −9.76 ± 13.32% vs. non-enhanced areas −7.18 ± 6.21%, *n* = 19) or fluidity (*p* = 0.1246, Gd-enhanced areas 4.47 ± 7.96% vs. rest of the brain 1.07 ± 10.56%, *n* = 19) (Figure 7B). However, when we compared the percentage change in stiffness between areas with Eu-VSOP accumulation and those without Eu-VSOP accumulation, we observed a significant difference (*p* = 0.0483, areas with Eu-VSOP accumulation −16.81 ± 16.49% vs. areas without accumulation −5.85 ± 3.81%, *n* = 8) (Figure 7B). This effect was also seen for global stiffness after establishment of EAE signs vs. stiffness at sites of Eu-VSOP accumulation (*p* = 0.0229, whole brain 2.93 ± 0.16 m/s vs. Eu-VSOP accumulation 2.55 ± 0.47 m/s, *n* = 8) (Supplementary Figure 4). This result indicates that, besides being sensitive to global brain changes, MRE can effectively identify areas affected by inflammatory processes that are detectable by Eu-VSOP accumulation only. Fluidity in these above mentioned locations, however, was unaffected (*p* = 0.2889, areas with Eu-VSOP accumulation −5.29 ± 8.33% vs. non-hypointense areas −2.16 ± 6.63%, *n* = 8) (Figure 7B).

### Visualization of Eu-VSOPs by IMC Reveals Association of Eu-VSOP Accumulation With Inflammatory Damage

**FIGURE 8:**
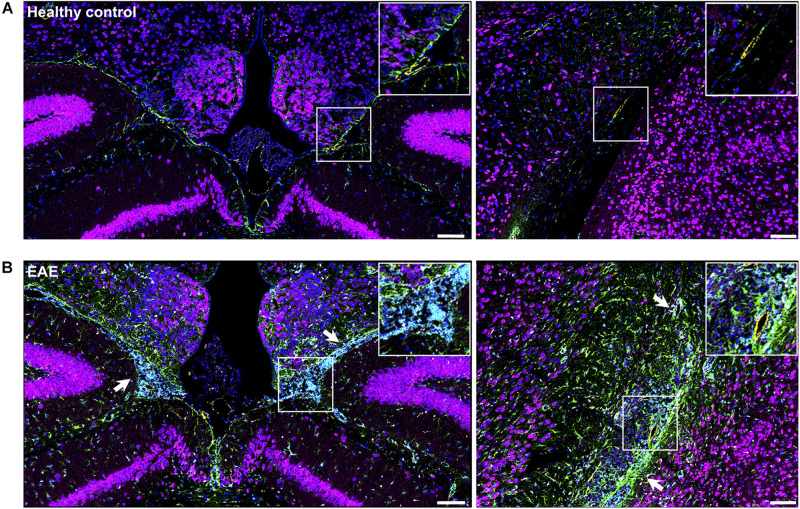
Visualization of brain inflammation induced by EAE using IMC. **(A)** Brain section of a healthy control (naïve) mouse at the third ventricles (left) and brain stem and hippocampal formation (right). **(B)** Extensive inflammation during EAE can be visualized by infiltration of CD45^+^ cells (cyan), astrogliosis (GFAP, green) and activated microglia (Iba-1, white) (white arrows). Neurons (NeuN—magenta), astrocytes (GFAP—green), endothelial cells (CD31—yellow), nuclei (histone H3—blue), microglia (Iba-1—white), leucocytes (CD45—cyan). Scale bar: 100 μm.

**FIGURE 9:**
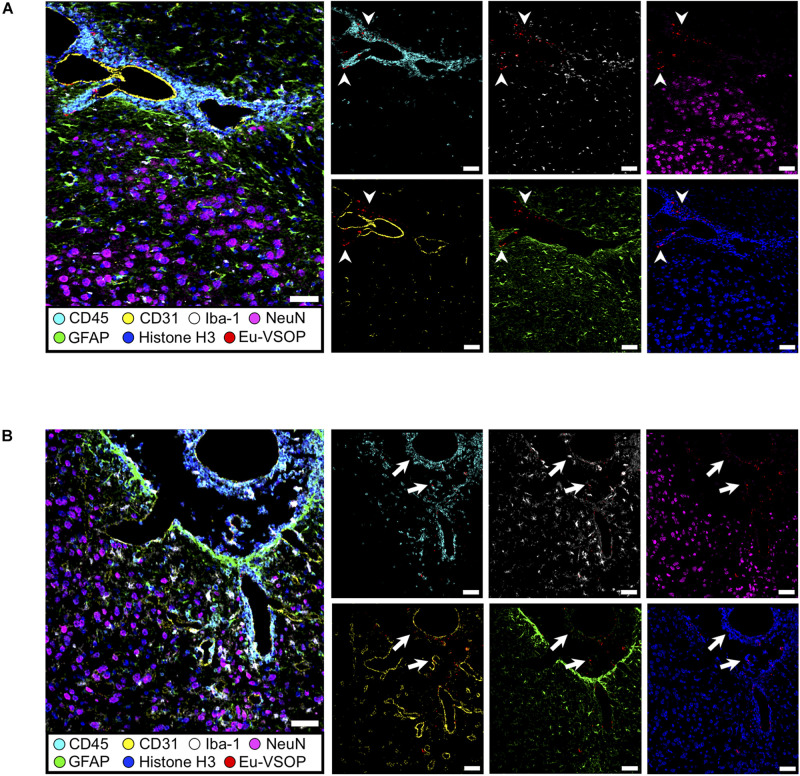
Histological visualization of Eu-VSOP distribution by IMC. **(A)** Magnetic particle accumulation in the perivascular space with strong leukocyte infiltrate (arrowheads) located between brain stem and hippocampal formation. **(B)** In the third ventricle, particles can be found in the choroid plexus and are associated with perivascular inflammation (arrows). Eu-VSOPs (red dots), neurons (NeuN—magenta), astrocytes (GFAP—green), endothelial cells (CD31—yellow), nuclei (histone H3—blue), microglia (Iba-1—white), leucocytes (CD45—cyan). Scale bar: 50 mm.

To confirm that the histopathological changes are indeed associated with inflammation-induced Eu-VSOP accumulation, we investigated inflammation in EAE and healthy control brain sections as well as the tissue distribution of Eu-VSOP and co-localization with different inflammatory and barrier cells using IMC (Figures 8, 9).

When compared with healthy control, EAE mice showed evident inflammation, with gliosis, represented by increase in Iba^+^(white) cells and GFAP (green), as well as infiltration of immune cells (CD45, cyan) (Figure 8, white arrows). In sites where Eu-VSOPs were visualized by MRI, the particles were found to be associated with sites of inflammatory damage. In particular, areas of Eu-VSOP accumulation presented CD45 + leukocyte infiltration, mostly concentrated in perivascular cuffs, in the periventricular space (Figure 9A, arrowheads) as well as in the choroid plexus (Figure 9B, white arrows). Iba-1 + cells (microglia, macrophages) were frequently localized alongside immune infiltrates. Perivascularly, Eu-VSOP accumulation was confined to these sites of injury and colocalized with Iba-1 + and CD45 + positive cells. Furthermore, Eu-VSOPs (red dots) were found to be especially associated to the vessel walls, where they were co-localized with activated endothelial cells (CD31+). In the ventricles, Eu-VSOPs (red dots) were confined to the choroid plexus, showing a clear association with the presence of immune infiltrates (Figure 9B). No particles were found to be co-localized with NeuN + neurons. Due to the IMC resolution of 1 μm and the particle dynamic diameter of 7 nm, quantification of the particle count could not be determined.

## Discussion

MRE has been previously shown to identify global changes in brain stiffness both in MS and in its animal model, EAE (Wuerfel et al., 2010; Streitberger et al., 2012; Riek et al., 2012; Millward et al., 2015; Wang et al., 2020). To further understand the neuropathological alterations detected by MRE, we combined MRE with GBCA- and Eu-VSOP-enhanced MRI. Tissue viscoelasticity was then evaluated in areas of BBB leakage (GBCA-enhancing areas) and in sites with Eu-VSOP accumulation, i.e., areas where we have previously identified activated myeloid cells and/or altered endothelium or epithelia. To the best of our knowledge, this study for the first time combines analysis of these biochemical imaging markers with MRE findings across multiple slices in a mouse model of neuroinflammation, revealing that areas of exclusive GBCA or Eu-VSOP signal displayed significant tissue softening. When these areas were compared with non-enhancing brain areas, stiffness changes at sites of Eu-VSOP accumulation showed more pronounced softening than areas of Gd-enhancement, suggesting that MRE is especially sensitive to detect inflammation-associated changes in tissue viscoelasticity. Subsequent histological visualization of Eu-VSOPs by IMC confirmed their distribution in areas of intense focal inflammation, likely undergoing significant tissue remodeling.

Here, brain viscoelastic properties were investigated in the adoptive transfer EAE model using SJL mice. The induction of this model, introduced by injection of a pre-activated population of myelin epitope-specific CD4 + T cells, results in a more severe disease with large cerebral infiltrations compared to active immunization, as used in previous studies (Riek et al., 2012; Wang et al., 2020). While in C57BL/6 mice, immunized with myelin oligodendrocyte glycoprotein (MOG) (Miller and Karpus, 2007), principally spinal cord lesions are detected, making this model less suitable for cranial MRE studies (Millward et al., 2015). Thus, the relapse-remitting adoptive transfer model in SJL mice is an appropriate tool for brain imaging studies.

In this study we confirm previous work reporting a significant global decrease in stiffness during neuroinflammation in both patients and mouse models of neuropathologies such as Alzheimer (Murphy et al., 2011; Munder et al., 2018), Parkinson disease (Lipp et al., 2013; Klein et al., 2014; Hain et al., 2016), and MS (Wuerfel et al., 2010; Streitberger et al., 2012) (Riek et al., 2012; Millward et al., 2015; Fehlner et al., 2016; Wang et al., 2020). Based on gene expression and histopathological investigations, this reduction in stiffness correlated with the severity of inflammation. In the EAE model, an increased expression of CD3, a T-cell lineage marker (Riek et al., 2012), and F4/80 gene expression, a marker for macrophages (Millward et al., 2015), positively correlated with brain softening. Furthermore, enhanced expression of the extracellular matrix glycoprotein fibronectin was correlated with decreased stiffness, hinting at a role of neurovascular remodeling at lesion sites that contributes to changes in mechanical tissue properties (Wang et al., 2020). However, previously MRE changes were only investigated in large brain areas within one slice and thus could not account for regionally resolved inflammation. By combining contrast-enhanced MRI and multifrequency MRE with tomoelastography post processing, we were able to directly measure changes in viscoelastic properties at sites of neuroinflammation. Although, MRE was limited to the same brain segment included in previous studies using tomoelastography (Bertalan et al., 2019; Bertalan et al., 2020; Guo et al., 2019), known for acquiring multifrequency wavefield for high-resolution MRE, we were able to acquire multiple slices ranging from bregma −2.84 to 0.23 and with a resolution sufficient to resolve areas of focal inflammation. This allowed us to register MRE maps to a reference atlas, thus making the comparison within areas of contrast agent enhancement possible.

GBCA-enhanced MRI visualizes BBB permeability not only in MS patients but also in EAE models (Hawkins et al., 1990; Nessler et al., 2007; Schellenberg et al., 2007; Smorodchenko et al., 2007; Tysiak et al., 2009; Wuerfel et al., 2007; Waiczies et al., 2012). To investigate the potential of MRE in identifying BBB-related pathological events, we directly compared mechanical properties of the brain and Gd distribution. Here, the quantification of viscoelastic parameters revealed a significant decrease in tissue stiffness in adoptive transfer EAE.

As apparent from the incidence maps, all mice had GBCA enhancement, as also shown by Smorodchenko et al., 2007. Confirming previous results (Smorodchenko et al., 2007; Wuerfel et al., 2007), our findings showed marked involvement of the brain, with extensive cerebellar enhancement (bregma −7.32 and −5.50 mm), notably at the 4th ventricle; similarly, but to a lesser extent, the brain stem was also affected. Cerebellar susceptibility to BBB breakdown is an expected outcome of this model and is attributable to the high degree of vascularization in this area (Tonra, 2002). Other regions showing Gd-enhancement included the periventricular areas, hippocampus, thalamus, hypothalamus and midbrain (bregma −3.26 to −1.86), with particularly pronounced enhancement close to major vessels such as the hippocampal artery and in their adjacency. This pattern of enhancement has been described in other EAE models before (Kuharik et al., 1988; Smorodchenko et al., 2007; Wuerfel et al., 2007).

However, Gd-enhancing areas showed a reduction in stiffness similar to that observed in non-enhancing areas of the inflamed brain. Given that Gd-enhancement is often visualized as areas of diffuse hyperintensity and much more extensive compared with the pattern of Eu-VSOP accumulation, we hypothesize that the viscoelastic effect observed in areas of Gd-enhancement does not reflect strictly localized histopathological alterations purely associated with BBB breakdown, but rather indicates predominantly a disseminated softening due to the inflammatory state of the brain. Although GBCA diffusion within the tissue primarily represents increased BBB permeability and has been correlated with glial cell activation (Nessler et al., 2007; Morrissey et al., 1996), its spatial distribution is not only limited to these events. After crossing the BBB, GBCA diffusion and dispersion throughout the tissue could also be attributed to factors such as the contrast agent’s distribution in the interstitial and intracellular spaces, an inherent property of a molecule’s biodistribution (Aime and Caravan, 2009), the degree of vascular disruption and leakiness (Layne et al., 2018), or the reduction of perfusion or extracellular space in areas of inflammatory lesions (Nessler et al., 2007).

Previous studies have shown that Gd-enhancing lesions are present before disease signs become apparent (Wuerfel et al., 2007) but rarely correlate with the presence of characteristic immune infiltrate-derived lesions, a hallmark of EAE and MS (Nessler et al., 2007; Nathoo et al., 2014). In contrast, we show that Eu-VSOPs also accumulate in areas that are not enhanced by GBCA and are present at sites of immune cell infiltration, which is in alignment with the literature (Tysiak et al., 2009; Millward et al., 2019; Berndt et al., 2017). More precisely, we found Eu-VSOPs to be dispersed and mostly located in the vicinity of vessels and ventricles, as indicated in the incidence maps. In the cerebellum, comprising approximately the bregma coordinates −7.32 to −5.50 mm, Eu-VSOP accumulations were found next to the 4th ventricle, the dorsomedial cerebellar arteries, lateral superior cerebral artery, and close to the basilar artery. In the bregma −3.26 mm to 0.23 mm, including structures such as the hippocampus, and the lateral and 3rd ventricles, Eu-VSOPs appear to be located close to the anterior choroidal artery (for anatomical references refer to Dorr et al. (2007) and Xiong et al. (2017). Different routes have been proposed to explain how VSOPs can enter the brain: phagocytosis by peripheral macrophages (Tysiak et al., 2009; Millward et al., 2019) and activated T cells (Wuerfel et al., 2007), which then infiltrate the CNS; passive diffusion through the leaky BBB as freely diffusing particles as observed in the parenchyma of EAE mice (Millward et al., 2013); and binding to endothelial or epithelial barriers (Plewes et al., 1995; Streitberger et al., 2012). Eu-VSOPs were imaged 24 h after injection when they have been shown to be cleared from the blood (Wuerfel et al., 2007). Therefore, we propose that hypointense signals seen in T2^∗^ images originate primarily from VSOP bound to altered ECM components (Berndt et al., 2017) or from particles phagocytosed by peripheral macrophages or/and activated microglia present at sites of inflammation (Tysiak et al., 2009; Millward et al., 2013, 2019). However, it cannot be excluded that these hypointense signals originate from particles phagocytosed by intravascular immune cells patrolling the endothelium and ready to cross upon inflammatory signals as reported in Masthoff et al., 2018 (Masthoff et al., 2018). Taken together, these evidence indicate that magnetic particles reveal histopathological alterations other than the well-known BBB disruption underlying GBCA enhancement (Tysiak et al., 2009; Millward et al., 2013; Berndt et al., 2017).

Importantly, our results show that, during inflammation, stiffness is particularly reduced in regions of Eu-VSOP accumulation compared to global brain softening. To elucidate underlying alterations in sites with Eu-VSOP accumulation that might contribute to the marked reduction in stiffness, we performed histological assessment of the particles. Differently from the unspecific method to detect iron particles, the Prussian blue stain, here we show that Eu-VSOPs can be visualized by IMC. The IMC allows a distinguishable detection of the Eu present in the core of the particles, and additionally, provides important information on the cellular environment in sites of particle accumulation. We found that Eu-VSOPs accumulated at sites of inflammation, as co-localization with leukocyte infiltrate was evident, in line with our previous reports (Millward et al., 2013). In areas without hypointense signal, particles may be found scattered in the tissue, possibly after diffusing through the disrupted BBB and accumulating in the brain parenchyma. Consequently, passively diffused particles would not contribute to the MRI signal 24 h after injection as much as the phagocytosed particles accumulated in sites of strong inflammation.

In contrast to tissue from EAE mice, histological analysis of healthy control further confirmed no Eu-VSOP accumulation in the non-inflamed brain tissue, indicating that Eu-VSOP accumulation is caused by processes of inflammation. Accumulations within the investigated inflamed tissue sections were mostly concentrated in the perivascular cuffs and in the periventricular space as well as in the choroid plexus. In areas of inflammatory alterations, we found particles co-localized with endothelial cells, which could have been uptaken *via* inflammation-induced alterations of sulfated glycosaminoglycans on the endothelial glycocalyx (Berndt et al., 2017) and with CD45 + and Iba-1 + cells, suggesting that they were phagocytosed. During EAE, astrogliosis and pronounced microglial activation were observed, presenting a typically hypertrophic and ramified morphology. Eu-VSOPs were also specifically co-localized with activated microglia, consistent with previous reports (Wuerfel et al., 2007; Tysiak et al., 2009; Millward et al., 2013, 2019). Atomic force microscopy has shown that glial cells are relatively softer than their neighboring neurons (Lu et al., 2006), as GFAP-expressing cells were associated with lower stiffness values (Moeendarbary et al., 2017). Therefore, in inflamed regions, the presence of softer glial cells could be one contributing factor to the marked decrease in stiffness observed in sites of Eu-VSOP accumulation. Additionally, the inflammatory activity of infiltrating peripheral immune cells and activated glial cells in these areas could result in extensive remodeling of the ECM. In addition, secretion of metalloproteinases during neuroinflammation leads to the degradation of ECM components, which facilitates further disruption of the BBB [for a review refer to Rosenberg (2002)]. A dynamic alteration of the ECM structure with accumulation of other matrix components, such as fibronectin, has also been demonstrated for EAE and correlates with neural tissue softening (Wang et al., 2020).

Moreover, although there are no studies directly associating vasogenic edema with viscoelastic changes in EAE, reports on mice with stroke lesions have linked edema with brain softening in the ipsilateral side (Xu et al., 2013, 2014). Thus, it is possible that vasogenic edema associated with the cellular inflammation depicted may also influence tissue elasticity in areas with VSOPs.

The pronounced softening observed at sites of Eu-VSOP accumulation suggests that an intense focal inflammatory process has a strong effect on viscoelastic parameters. This could be attributable to a combined influence of activated glial cells, accumulation of immune cells, local edema, and/or remodeling of the extracellular matrix, especially in the microenvironment of the neurovascular unit. On the other hand, GBCA-enhanced MRI reflects a transient event of enhanced vessel permeability and may only account for one aspect of the inflammatory state in a multifactorial disease model. Hence, the contribution of the dynamic disturbance of the BBB possibly results in a different distribution of Eu-VSOP accumulation compared to Gd-enhancement.

To be able to distinguish neuroinflammatory processes visualized either by GBCA or by VSOPs, masks comprising solely each contrast agent were generated. Areas, in which Eu-VSOP accumulation overlaps with Gd-enhancement might contain particles that just passively diffuse into the brain parenchyma through a damaged BBB. Thus, particles co-localizing with GBCA-enhancement would not reveal distinct aspects of inflammation different from BBB leakage and were not considered for this investigation.

One limitation of our study is that the brain area covered by tomoelastography is limited to the bregma areas −2.84 mm to 0.23 mm. It is well documented, for instance, that the cerebellum is a particularly affected region in EAE models (Tonra, 2002; Wuerfel et al., 2007; Millward et al., 2015) though it could not be included in our analysis. Therefore, it would be important to include these regions in future studies to incorporate crucial pathological information into the imaging findings. Moreover, based on previous studies, we consider that 24 h after injection, Magnevist is completely depleted from the blood and the brain (Tysiak et al., 2009; Wang et al., 2020). Thus, an effect on the T2^∗^-weighted image used for quantification of Eu-VSOP is highly unlikely. However, since we did not perform a T1 scan before the second T2^∗^-weighted scan, it is not possible to completely exclude a residual effect of the GBCA.

A noteworthy point to mention is that studies involving *in vivo* VSOP imaging requires intravenous administration of particles with the obvious associated safety concerns if a clinical application in the future is intended. As changes in viscoelastic properties in areas of VSOP accumulation was shown to be detectable by MRE, we propose that MRE may represent a suitable non-invasive imaging method that allows *in vivo* visualization of broad pathological processes. MRE could therefore be applied to both, pre-clinical and clinical settings in the context of neuroinflammation, serving as a tool for research, diagnostic, and therapy monitoring.

## Conclusion

Combining for the first time Gd-enhancement and Eu-VSOP with *in vivo* multifrequency MRE, we obtained results suggesting that MRE is able to detect non-invasively pathological processes associated with inflammation beyond BBB disruption and vascular leakage. Such processes visualized by Eu-VSOP-enhanced MRI may involve gliosis and macrophages infiltration as well as alterations of endothelial matrix components and ECM remodeling. MRE may serve to visualize *in vivo* a broad spectrum of pathological processes and represents, therefore, a useful non-invasive imaging tool for neuroinflammation in preclinical and clinical settings.

## Data Availability Statement

The raw data supporting the conclusions of this article will be made available by the authors, without undue reservation.

## Ethics Statement

The animal study was reviewed and approved by Berlin State Office for Health and Social Affairs (LAGeSo), Berlin, Germany.

## Author Contributions

AM and RS: experimental design, data acquisition, data analysis and interpretation, and manuscript writing. YR-S and DK: IMC design, image acquisition, image processing, and manuscript revision. HT: supervision of MRE data analysis and manuscript revision. AK: IMC resources and manuscript revision. JS and MT: Eu-VSOP resources and manuscript revision. SK: technical assistance, supervision of MRI data registration, and manuscript revision. SM and PB-S: technical assistance, supervision of MRI design and data analysis, and manuscript revision. CI-D and IS: conceptualization, data interpretation, funding acquisition, resources, methodology, project administration, supervision, and editing and critical revision of manuscript. All authors fully qualify for authorship and have approved the final version of the manuscript.

## Conflict of Interest

The authors declare that the research was conducted in the absence of any commercial or financial relationships that could be construed as a potential conflict of interest.

## Publisher’s Note

All claims expressed in this article are solely those of the authors and do not necessarily represent those of their affiliated organizations, or those of the publisher, the editors and the reviewers. Any product that may be evaluated in this article, or claim that may be made by its manufacturer, is not guaranteed or endorsed by the publisher.

## Footnotes

1
https://github.com/ChariteExpMri/antx2

2
http://elastix.isi.uu.nl/

3
https://imagej.nih.gov/ij/
