# Supplementary material for: Contribution of Tissue Inflammation and Blood-Brain Barrier Disruption to Brain Softening in a Mouse Model of Multiple Sclerosis
Source: Front Neurosci. 2021 Aug 23;15:701308. doi: 10.3389/fnins.2021.701308 (PMC8419310; doi:10.3389/fnins.2021.701308)
Supplement: Supplementary file 1 [file Table_1.docx]

Supplementary Material


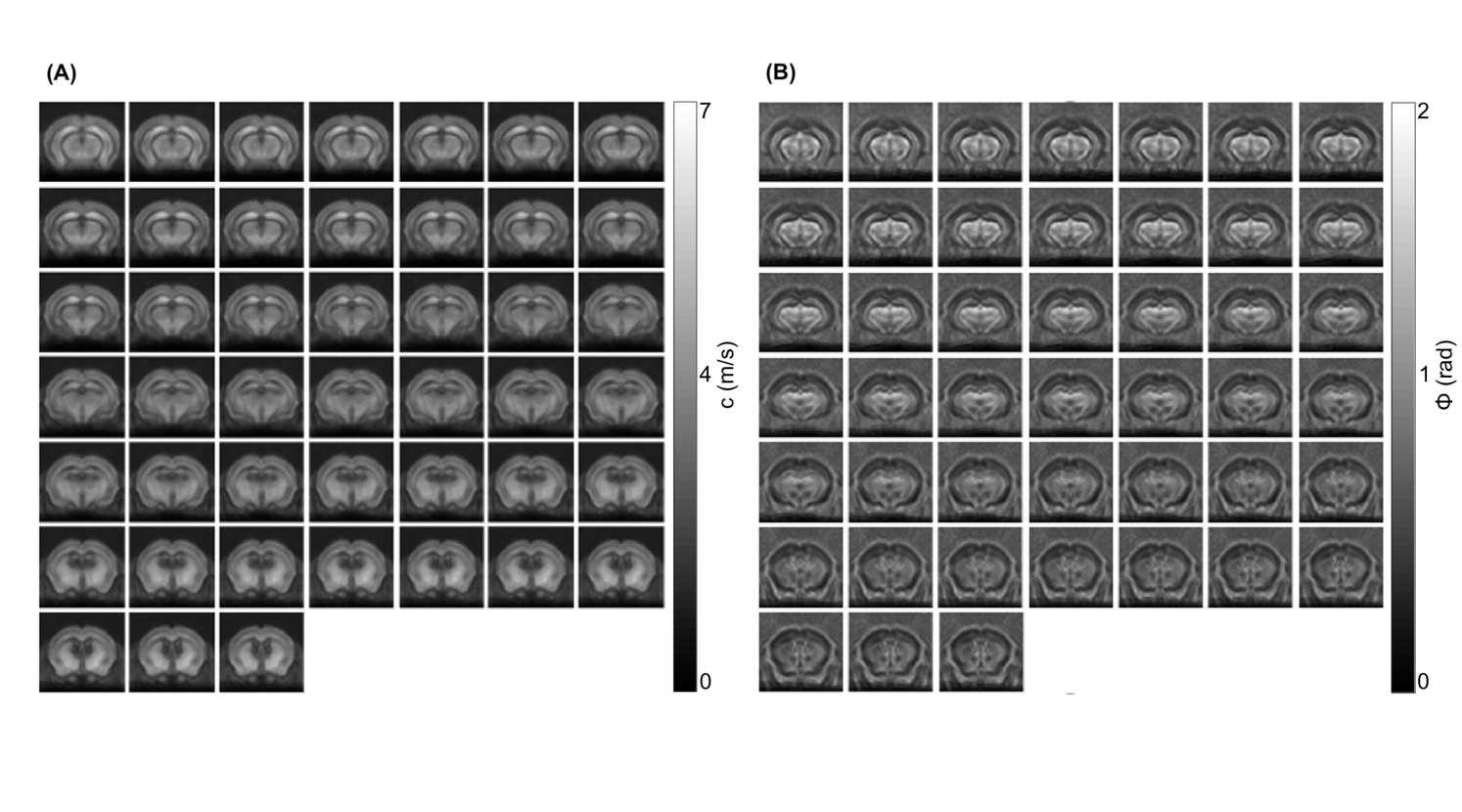


**Supplementary Figure 1: MRE parameter maps.** Bregma area -2.84 mm to 0.23 mm was covered by MRE showing an averaged A) stiffness map (c in m/s) and B) fluidity map (ϕ in rad).


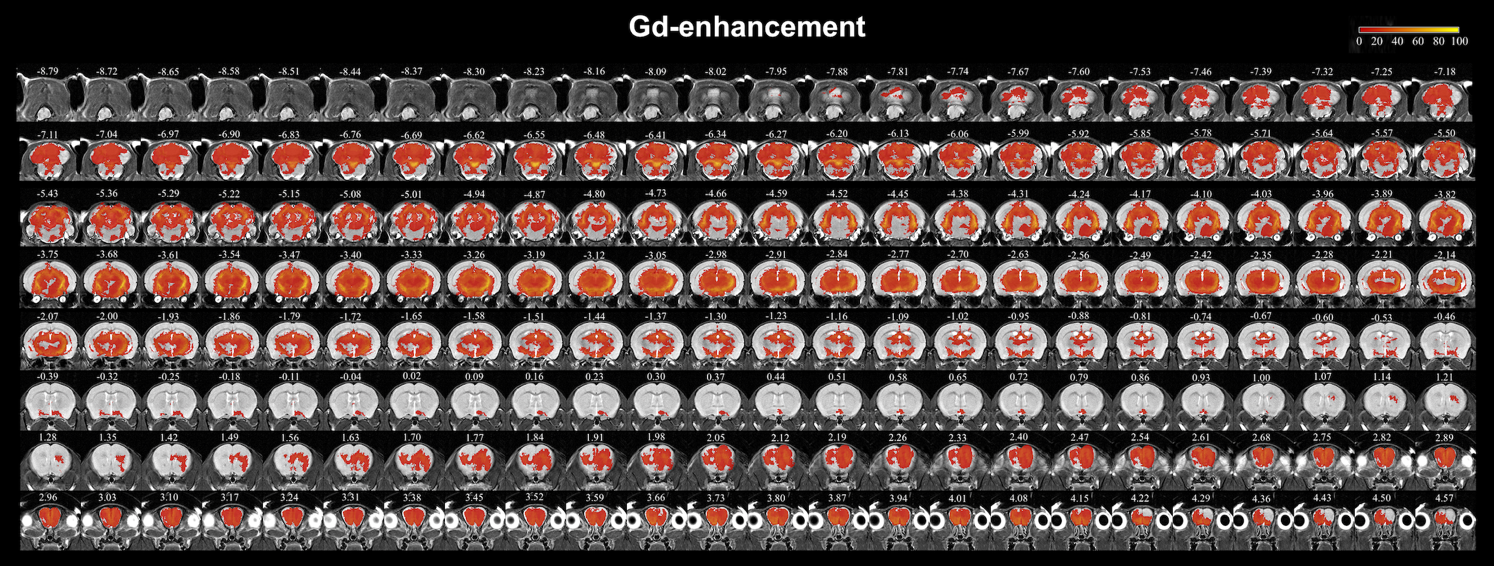


**Supplementary Figure 2:** **Incidence map of Gd-enhancement during EAE.** Overview of the incidence (%) of Gd-signal enhancement over the entire brain (*n* = 19).


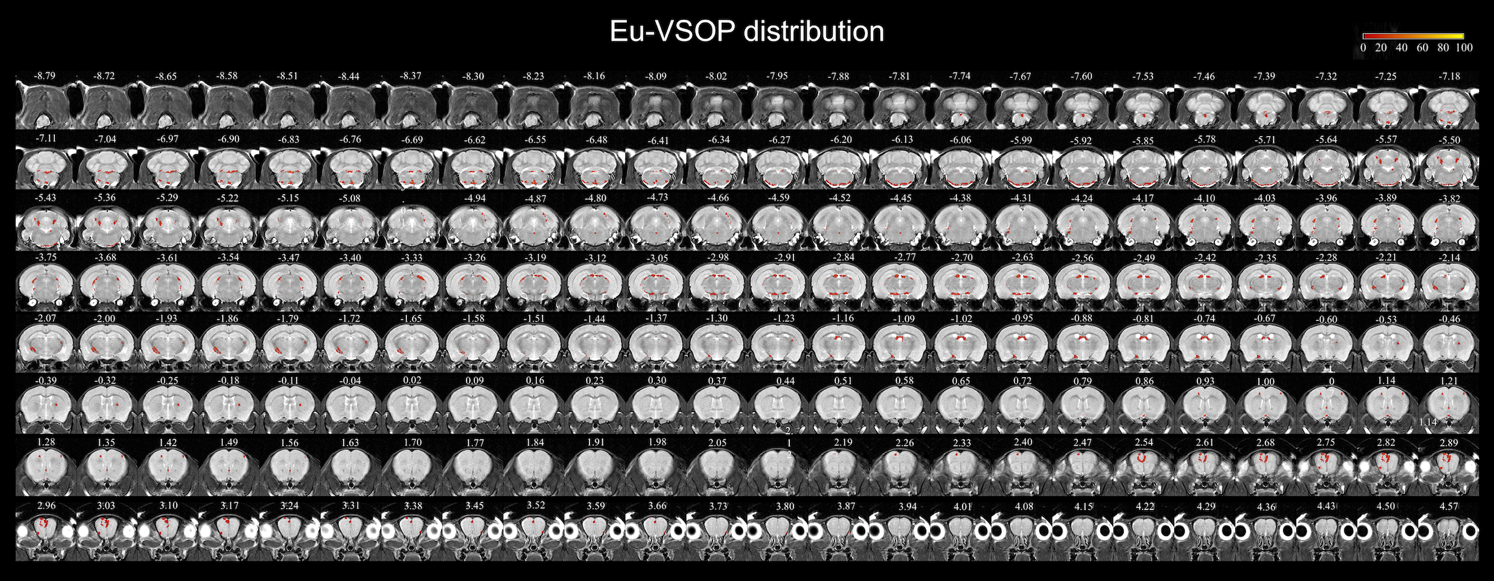


**Supplementary Figure 3:** **Incidence map of Eu-VSOP distribution during EAE.** Overview of the incidence (%) of signal loss on T2*w images representing Eu-VSOP accumulation over the entire brain (*n* = 11).

| **Antibody** | **Clone** | **Manufacturer** | **Isotope tag** | **Dilution** |
| --- | --- | --- | --- | --- |
| anti-NeuN | 1B7 | Biologend | 165Ho | 1:400 |
| anti-GFAP | EPR1034 | Abcam | 169Tm | 1:800 |
| anti-Iba-1 | EPR16589 | Abcam | 144Nd | 1:800 |
| anti-CD31 | D8V9E | Cell Signaling Technology | 164Dy | 1:800 |
| anti-CD45 | D3F8Q | Abcam | 166Er | 1:400 |
| anti-histone H3 | D1H2 | Fluidigm | 176Yb | 1:4000 |

**Supplementary Table 1:** Overview of isotope-tagged antibodies.


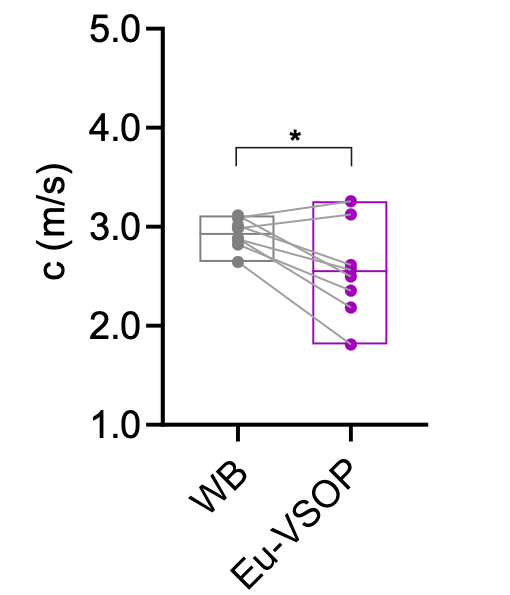


**Supplementary Figure 4:** Regions with Eu-VSOP accumulation are significantly softer than the whole brain after the establishment of EAE (p = 0.0229; whole brain: 2.93 ± 0.16 m/s vs. Eu-VSOP: 2.55 ± 0.47 m/s), *n* = 8, mean, min/max; *< 0.05.
